# Comparison of Melphalan Combined with Treosulfan or Busulfan as High-Dose Chemotherapy before Autologous Stem Cell Transplantation in AML

**DOI:** 10.3390/cancers14041024

**Published:** 2022-02-17

**Authors:** Ekaterina Gurevich, Michael Hayoz, Yolanda Aebi, Carlo R. Largiadèr, Behrouz Mansouri Taleghani, Ulrike Bacher, Thomas Pabst

**Affiliations:** 1 Department of Medical Oncology, Inselspital, University of Bern, CH-3010 Bern, Switzerland; ekaterina.gurevich@students.unibe.ch; 2 Department of Clinical Chemistry, Inselspital, University of Bern, CH-3010 Bern, Switzerland; michael.hayoz@insel.ch (M.H.); yolanda.aebi@insel.ch (Y.A.); carlo.largiader@insel.ch (C.R.L.); 3 Center of Laboratory Medicine (ZLM), Inselspital, University of Bern, CH-3010 Bern, Switzerland; 4 Department of Hematology, Inselspital, University of Bern, CH-3010 Bern, Switzerland; behrouz.mansouritaleghani@insel.ch (B.M.T.); veraulrike.bacher@insel.ch (U.B.)

**Keywords:** acute myeloid leukemia (AML), autologous stem cell transplantation (ASCT), high-dose chemotherapy (HDCT), treosulfan, biovariability, pharmacologic monitoring

## Abstract

**Simple Summary:**

Different consolidation strategies are available for acute myeloid leukemia (AML) patients fit for intensive treatment. For favorable- or intermediate-risk AML, high-dose chemotherapy (HDCT) followed by autologous stem cell transplantation (ASCT) is one of these options. Busulfan plus melphalan is a frequently used and efficient HDCT regimen, but it bears neurotoxic potential and may cause irreversible alopecia, amongst other toxicities. Thus, improving HDCT regimens with lesser toxicity, albeit at comparable anti-leukemic efficacy, is wishful. We combined treosulfan with its more favorable toxicity profile with melphalan for HDCT and compared these patients with a group receiving busulfan/treosulfan. Whereas disease-free and overall survival did not differ significantly, the treosulfan regimen compared favorably, with the absence of neurotoxicity and irreversibly alopecia. Treosulfan serum levels by mass cytometry demonstrated considerable interindividual biovariability. Further studies should explore treosulfan/melphalan for HDCT/ASCT in AML, aiming to improve the quality of life of AML survivors and offer safer consolidation strategies.

**Abstract:**

(1) Background: High-dose chemotherapy (HDCT) before autologous stem cell transplantation (ASCT) in acute myeloid leukemia (AML) patients predominantly combines busulfan with cyclophosphamide or melphalan. Treosulfan compares favorably regarding lower inter-individual bioavailability and neurotoxicity, but so far, had not been studied before ASCT in AML. (2) Methods: This single-center study investigated AML patients undergoing ASCT in CR1 between November 2017 and September 2020. The first 16 patients received busulfan 16 mg/kg b.w. (days −5 to −2) and melphalan 140 mg/m^2^ (day −1) (BuMel). In a subsequent (TreoMel) cohort, 20 patients received treosulfan 14 g/m^2^ (days −4 to −2) and melphalan. Plasma concentrations of busulfan and treosulfan were determined by mass spectrometry. (3) Results: Neutrophil engraftment and platelet recovery were similar, and PFS and OS were comparable. In only the BuMel cohort, patients reported central nervous toxicities, including seizures (6%) and encephalopathy (12%). The mean AUC for busulfan was 1471.32 μM*min, and for treosulfan it was 836.79 mg/L*h, with ranges of 804.1–2082 μM*min and 454.2–1402 mg/L*h. The peak values for busulfan ranged between 880.19–1734 μg/L and for treosulfan between 194.3–489.25 mg/L. (4) Conclusions: TreoMel appears to be safe and effective for pre-ASCT treatment in AML patients. Due to considerable interindividual biovariability, pharmacologic monitoring may also be warranted for the use of treosulfan.

## 1. Introduction

Curative treatment in patients with acute myeloid leukemia (AML) consists of intensive induction and consolidation therapy, aiming to achieve hematological remission after induction therapy and to prevent relapse by consolidation treatment. Based on molecular and cytogenetic risk assessment conducted at the time of diagnosis and response to induction treatment, various consolidation therapy options are considered [1]. For good- and intermediate-risk patients, high-dose chemotherapy (HDCT) followed by autologous hematologic stem cell transplantation (ASCT) or chemotherapy consolidation are options, whereas the standard approach for adverse risk or MRD-positive, intermediate-risk patients is allogeneic stem cell transplantation [2,3,4,5]. HDCT/ASCT in MRD-negative and good- or intermediate-risk patients is effective and prolongs survival, with less toxicities as compared to allogeneic stem cell transplantation [2,4,5,6,7,8,9]. This may be particularly beneficial in older patients [2].

Historically, the most common conditioning regimen used for HDCT before ASCT was busulfan at a total dose of 16 mg/kg combined with cyclophosphamide at a total dose of 120 mg/kg (BuCy) [10]. However, in 2018, a large retrospective study of the European Society for Blood and Marrow Transplantation (EBMT) concluded that busulfan combined with melphalan (BuMel) resulted in higher overall survival (OS) rates compared to BuCy after ASCT [10].

Whereas the BuMel combination is obviously effective, busulfan treatment is associated with considerable toxicity, ranging from inducing severe emesis and irreversible alopecia to CNS toxicity with seizures [11,12,13,14]. Another serious side effect is veno-occlusive disease, which was reported in 7% to 28% of patients [12,13,14,15,16]. Moreover, considerable interindividual differences in the bioavailability of busulfan requires monitoring of serum levels with eventual dose modification. Finally, busulfan is metabolized through glutathione conjugation, which may lead to a number of clinically significant interactions with other drugs metabolized through this pathway [12,16]. In conclusion, alternatives to busulfan may provide clinical advantages in many aspects.

Treosulfan offers a more favorable safety profile compared to busulfan. Its antileukemic effect together with cyclophosphamide (TreCy) was shown to be as effective as BuCy [15,17], while no veno-occlusive disease was reported [18]. In the allogeneic transplant setting, Beelen et al. investigated the use of treosulfan in combination with fludarabine, in higher risk and (mostly) elderly patients with MDS and AML, as a conditioning regimen before allogeneic stem cell transplantation, and the authors reported improved event-free and overall survival in patients receiving treosulfan-based conditioning compared to a busulfan-based conditioning regimen. This was due to a lower transplant-related mortality, which was eventually related to the stronger immunosuppressive profile of treosulfan as compared to busulfan through the activation of pro-apoptotic protein kinase C, induced caspase activation, and downregulation of antiapoptotic mcl-1 protein [11,12,15,19,20]. Finally, treosulfan was shown to reduce lymphocyte counts for longer periods, resulting in suppressed cytokine production (specifically, IL-2 and INF-γ), which is involved in the development of subsequent graft versus host disease (GvHD) [12]. Treosulfan is a prodrug of a bifunctional alkylating agent, and it has a high pH- and temperature-sensitive nonenzymatic conversion to the active agent [21,22]. The elimination of the drug occurs through dose-dependent glomerular filtration with a tubular reabsorption rate of around 60%, with other ways of elimination also being involved [18,21]. Treosulfan offers a relatively short half-life of 1.8 h [16,23,24]. The debate is ongoing whether treosulfan shows interpatient variability of distribution at a steady state (V_ss_), either due to differences in total body water percentage or age, and whether the AUC correlates with the toxic effects, as shown for treosulfan [21,25,26].

So far, treosulfan has not been investigated as a conditioning treatment before ASCT. In this study, we report the experiences in a subsequent cohort of AML patients treated with treosulfan combined with melphalan (TreoMel) as consolidation treatment for AML patients in first remission before ASCT. We also performed a comparison to the busulfan/melphalan combination (BuMel) before ASCT. Additionally, we performed analysis of plasma concentrations of treosulfan by mass spectrometry to investigate the bioavailability of the drug, and to compare it to busulfan.

## 2. Materials and Methods

### 2.1. Patients

This is a single-center retrospective study comparing two consecutive cohorts. We analyzed consecutive patients with favorable- or intermediate-risk (MRD-negative) AML fit for intensive treatment, who had reached MRD-negative CR following two cycles of induction chemotherapy using the standard protocol of an anthracycline and cytarabine agent, and who received HDCT either with BuMel or with TreoMel followed by ASCT. MRD was examined using flow cytometry in all patients, and when suitable molecular markers were available, such as reciprocal rearrangements or NPM1 mutations (frequent subtypes), quantitative real-time PCR (qPCR) was added. All patients who complied with the abovementioned criteria and were diagnosed with AML between 2017 and 2020 at the University Hospital Bern were included in the study. More information on patient inclusion can be found in Supplementary Figure S1. At the time of the start of consolidation treatment, all patients were in CR. All patients were treated at the University Hospital of Bern, Switzerland, and they have given written informed consent. The study was approved by the local ethics committee of Bern, Switzerland (decision number 2020-01503).

### 2.2. Treatment

For induction therapy, patients intravenously received cytarabine 200 mg/m^2^ on days 1 to 7 and idarubicine 12 mg/m^2^ on days 1 to 3 in cycle 1; and cytarabine 1000 mg/m^2^/q12h on days 1 to 6 and daunorubicin 60 mg/m^2^ on days 1, 3, and 5 were given in cycle 2. Mobilisation of peripheral blood stem cells (PBSC) was done in the regeneration period following induction chemotherapy, alongside the use of granulocyte colony-stimulating factor (G-CSF).

The first cohort of AML patients was treated with the BuMel regimen and the second cohort of patients with TreoMel. The BuMel combination was given to patients in CR between November 2017 and July 2019, and TreoMel was given to patients in CR from August 2019 to September 2020. All patients were treated at the same institution. Patients receiving either BuMel or TreoMel must have had favorable- or intermediate-risk AML, whereas adverse-risk AML patients were included if allogeneic transplantation was not possible for any reason.

Patients treated with BuMel received 1 mg/kg of busulfan p.o. every 6 h starting with day −5 of treatment to day −2, for a total of 16 mg/kg. This was followed by melphalan 140 mg/m^2^ on day −1 and ASCT on day 0. Patients treated with TreoMel received treosulfan 14 g/m^2^/day on days −4 to −2, followed by melphalan 140 mg/m^2^ on day −1 and ASCT on day 0. Treosulfan was administered intravenously in 1000 mL glucose 5% over a period of 2 h. Melphalan was administered in 500 mL NaCl 0.9% intravenously through a central venous catheter over one hour.

### 2.3. Supportive Therapy

Antiemetic therapy consisted of aprepitant, ondansetron, metoclopramide, and methylprednisolone. Additionally, all patients received pantoprazole prophylaxis. Oral cryotherapy was used during melphalan infusion to prevent mucositis. Excessive hydration during melphalan infusion was corrected using furosemide. Antiallergic prophylaxis before ASCT was performed by methylprednisolone and clemastine. Prophylaxis of hyperuricemia by allopurinol was continued over 7 days. All patients received enoxaparin (Clexane^®^ 40 mg s.c.) to prevent sinusoidal obstruction syndrome (SOS). Folic acid was administered to improve hematologic recovery for 8 weeks post-ASCT. Antibiotic prophylaxis consisted of sulfamethoxazole 800 mg/trimethoprim 160 mg, while the virostatic prophylaxis was valaciclovir 500 mg. In the BuMel cohort only, patients received antiepileptic prophylaxis with phenytoin.

### 2.4. Assessment of Adverse Events and Survival Rates

Adverse events were collected from daily follow-up reports by both physicians and nursing staff, as well as lab reports and microbiology reports. Severity was assessed using version 5.0 of the Common Terminology Criteria for Adverse Events (CTCAE) by the National Cancer Institute [27]. The comparison focused on non-hematologic adverse events, but hematologic parameters, such as the duration of cytopenias, were also collected. Progression-free survival (PFS) and overall survival (OS) were assessed based on reports from the follow-up visits of patients in the outpatient department of the clinic.

### 2.5. Plasma Concentration Measurement

Assessment of busulfan (given every six hours) concentration was performed after the fifth administration. On the second day of treosulfan treatment, blood samples were taken from all patients at the start of injection, after 30 min, and after 1, 2, 4, and 6 h to allow serial assessment of treosulfan concentration in the peripheral blood using an ultra-performance liquid chromatography chromatography-tandem mass spectrometry method (UPLC-LC-MS/MS). The mass spectrometric measurements were performed by multiple reaction monitoring on a Xevo TQ-S (Waters Corp., Milford, MA, USA). Samples were stabilized immediately during the collection at the clinical sites by the addition of a sodium citrate buffer to lower the pH and then stored at −80 °C until analysis.

Separate solutions of the following drugs and the isotope-labeled analogue were prepared: treosulfan and the internal standard (^2^H_4_)-treosulfan (w = 95%; Alsachim; Illkirch Graffenstaden, France) were directly solved in methanol at a concentration of 2 mg/mL. A mixed stock solution of non-deuterated compounds at 180 mg/L for treosulfan and 13 mg/L for busulfan in methanol were used for the preparation of calibrators. In the same way, an independent mixed stock solution was prepared for the quality controls.

Six calibrator-spiking solutions were prepared by diluting the stock solutions with methanol to final concentrations of 2.8, 5.6, 11.3, 22.5, 45, and 90 mg/L for treosulfan and 0.2, 0.4, 0.8, 1.6, 3.3, and 6.5 mg/L for busulfan. The same procedure was repeated for three quality control-spiking solutions, with the final concentrations of 4.2, 17, and 56 mg/L for treosulfan and 0.3, 1.2, and 4.1 mg/L for busulfan in methanol. Furthermore, a mixed internal standard stock solution containing 30 mg/L treosulfan and 2.0 mg/L busulfan was prepared in methanol.

The daily used working solution for the precipitation was prepared by diluting the internal standard stock solution to 1:20 with acetontrile (*v/v*).

Prior to analysis of treosulfan, the samples were prediluted to 1:20 with DC Mass Spect Gold serum (Golden West Biologicals, Temecula, CA, USA). For protein precipitation and analyte extraction of calibrators and quality controls, 25 µL of the calibrator- and quality control-spiking solutions, at the appropriate concentration, followed by 180 µL acetonitrile containing the internal standards ((^2^H_4_)-treosulfan and (^2^H_8_)-busulfan; Sigma-Aldrich; Buchs, Switzerland) were added to 40 µL DC Mass Spect Gold serum (Golden West Biologicals). After incubation and mixing for 10 min, the samples were centrifuged at 4000 rcf at 20 °C for 15 min, and 80 µL supernatant was diluted to 160 µL with methanol containing 3% formic acid. The prepared samples were sealed and stored in the autosampler at 10 °C until analysis.

For UHPLC-MS/MS analysis, 0.5 µL of the prepared samples were injected into a reverse-phase CORTECS UPLC T3 column of 120 Å, 1.6 µm, and 2.1 mm × 100 mm (Waters Corp., Milford, Massachusetts, USA), with a gradient mobile phase comprising 0.1% ammonium acetate with 1% formic acid (A) and methanol containing 0.1% ammonium acetate with 1% formic acid (B). Each sample was resolved for 3.0 min at a starting flow rate of 0.385 mL/min with the linear gradient for 0–1.0 min from 5 to 60% B, followed by 98% B for 0.55 min at a flow rate of 0.385 mL/min and 0.25 min at a flow rate of 0.650 mL/min. From 1.80–3.0 min, conditioning was performed with 5% B at a flow rate of 0.5 m/L. At the end, the flow rate was reset to 0.385 mL/min for the next injection. The column temperature was 45 °C. The eluent was introduced by electrospray ionization into the mass spectrometer (Xevo TQ-S, Waters Corp., Milford, Massachusetts, USA), operating in positive ion electrospray ionization mode (ESI+). The capillary voltage was set to 500 V and the source offset to 50 V. The dissolving gas flow was set to 1200 L/h and the temperature to 650 °C. The cone gas flow was 250 L/h, and the source temperature was set to 150 °C. To establish the appropriate multiple reaction monitoring (MRM) conditions for the individual compounds, the cone voltage was optimized to maximize the intensity of the protonated molecular species (M + H)+ and the collision energy (eV) was adjusted to optimize the signal for the most abundant product ions, which were subsequently used for MRM analysis (Table S2).

Data processing was performed with TargetLynx, available in the MassLynx software (version 4.1, Waters Corp.) by integration of the area under the specific MRM chromatograms in reference to the integrated area of the isotope-labeled analogue. Calibration curves were constructed related to the concentration of patient samples in the range of 35–1130 mg/L treosulfan or 0.13–4.0 mg/L busulfan by linear regression with a weighting factor of 1/x.

### 2.6. Statistical Analysis

Overall survival (OS) was defined as the time from autograft to death, regardless of cause, or at last follow-up in patients still alive. Progression-free survival (PFS) was defined as survival with no evidence of relapse or progression. Relapse was defined as increase to more than 5% bone marrow blasts (cytomorphologic relapse) or occurrence of molecular detectable disease (MRD positivity) in the peripheral blood or bone marrow. The day of ASCT was considered as day 0, and data cut-off was 26 January 2021. Survival was calculated according to Kaplan-Meier, and survival outcomes were compared by log rank.

## 3. Results

### 3.1. Patient Characteristics and Treatment

36 patients were included in this study. The first cohort comprised 16 patients receiving BuMel, and the subsequent cohort of 20 patients received TreoMel as described above, followed by ASCT. All patients had reached CR after two cycles of induction treatment, and disease was not detectable by either flow cytometry or by molecular diagnostics according to the ELN criteria. Patients receiving BuMel or TreoMel had either favorable-risk (*n* = 17, 47%) or intermediate-risk (*n* = 15, 42%) AML (ELN criteria). Four patients (11%) had adverse-risk and received HDCT/ASCT due to the unavailability of an HLA-matched donor (three patients) or due to patient preference (one patient).

The characteristics of patients and disease at diagnosis are summarized in Table 1 and Supplementary Table S1. Both patient groups had comparable median age at diagnosis. The male/female ratio was balanced in the BuMel group, whereas there was a male preponderance in the TreoMel group. All patients received two cycles of induction chemotherapy consisting of high-dose cytarabine and an anthracycline.

### 3.2. Hematologic Recovery

The median neutrophil engraftment (defined by an absolute neutrophil count (ANC) above 0.5 × 10^9^/L) was documented in BuMel patients at day +14 after ASCT (range of 11 to 33 days) compared to day +13 (range of 10 to 21 days) in TreoMel patients (*p* = 0.42). The median platelet recovery (above 20 G/L) was documented at a median of day +43 and +30, respectively (range of 13 to 137 days and 11 to 89 days, respectively; *p* = 0.221). These data are presented in Table 2.

### 3.3. Infections during Hospitalization

All patients treated with TreoMel developed at least one febrile episode (>38 °C) during neutropenia. At least one causative agent was identified in 14 (70%) patients, mostly bacterial infection (*n* = 13, 65% of patients), and predominantly coagulase-negative staphylococci, escherichia coli, enterococcus faecium, or klebsiella pneumoniae. In two patients (10%), a viral infection (rhinovirus, COVID-19) was identified, and a fungal infection occurred in one patient (5%). This was comparable to the BuMel cohort, where all patients developed febrile neutropenia. At least one causative agent was identified in 12 (75%) patients. A bacterial germ was identified in 12 patients, with a comparable spectrum of identified germs as seen in the TreoMel cohort. A viral infection affected 19% (*n* = 3) of the patients, and one patient (6%), again, had a fungal infection. These data are summarized in Table 3.

### 3.4. Other Non-Hematologic Toxicities during Hospitalization

A comprehensive overview summarizing all observed toxicities is shown in Supplementary Table S2. The most relevant toxicities are listed in Table 4. When both treatment groups were compared, irreversible alopecia was found in seven (45%) patients in the BuMel group, whereas no patients in the TreoMel group developed irreversible total or partial alopecia (*p* = 0.0014).

In addition, central nervous system toxicities (encephalopathy and seizures) were not observed in the TreoMel group, while encephalopathy was seen in two (12%) BuMel patients, and seizures occurred in one patient (6%). Due to the low number of patients, these differences were not significant (seizures: *p* = 0.444; encephalopathy: *p* = 0.191).

Other toxicities did not differ between both treatment groups. The presence of veno-occlusive disease (VOD) was based on the modified Seattle criteria [27]. No cases of VOD were noted in either cohort. Laboratory findings showed increased liver enzymes in most patients in both cohorts (BuMel: *n* = 16/16; 100%; TreoMel: *n* = 18/20; 90%; *p* = 0.999). Finally, rates of engraftment syndrome [28] at the time of neutrophil engraftment were similar in both treatment groups, occurring in five BuMel patients (31%) and in four TreoMel patients (20%; *p* = 0.47).

### 3.5. Treosulfan Plasma Concentration and Correlation with Non-Hematologic Toxicities

The mean AUC for busulfan was 1471.32 ± 161.97 μM*min and for treosulfan, 836.79 ± 117.27 mg/L*h, with ranges of 804.1–2082 μM*min and 454.2–1402 mg/L*h, respectively. Peak values for busulfan ranged between 880.19 and 1734 μg/L (mean 1351 ± 108.2 μg/L) and for treosulfan between 194.3 and 489.25 mg/L (mean 317.05 ± 36.87 mg/L). The AUC for busulfan was comparable to data previously reported by others, with the therapeutic AUC window reported to be between 900 and 1500 μmoL/min [29]. The AUC for treosulfan described in previous reports is between 836.79 and 2400 mg/L*h. Accordingly, AUC values in this study lie within the range described in the literature. A summary of the AUC for treosulfan, summarizing different stud ies, is given in Table 5.

**Figure 1:**
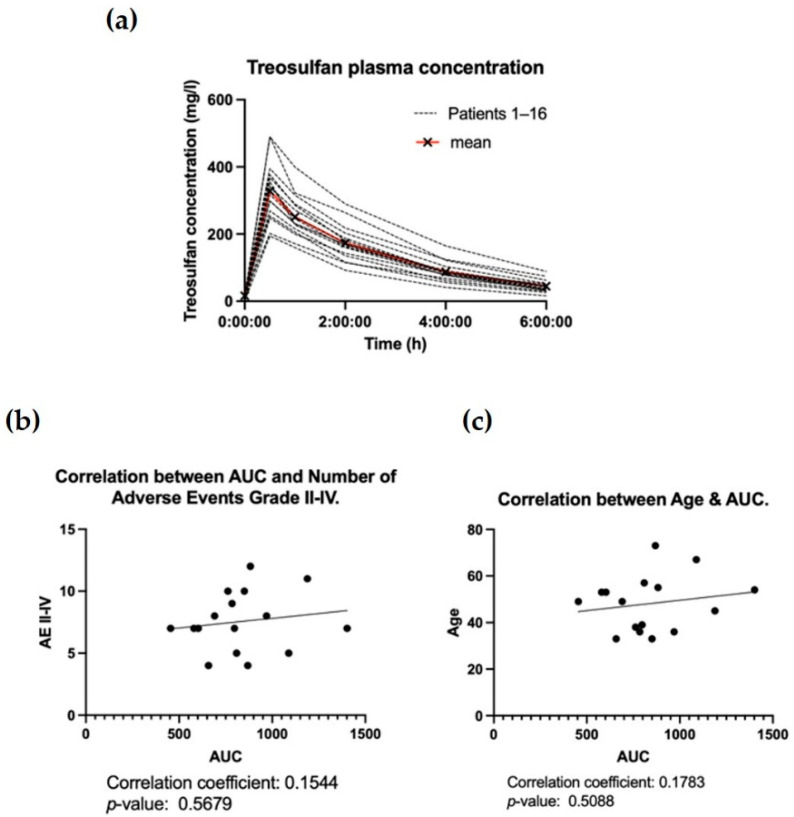
(**a**) Treosulfan plasma concentration. (**b**) Correlation between the AUC of the treosulfan group and the number of grade II–IV adverse events. (**c**) Correlation between patient age and AUC of the treosulfan treatment group.

We found a non-significant correlation between the AUC of treosulfan and the number of grade II–IV adverse events (correlation coefficient: 0.1544, *p*-value: 0.568). Similarly, there was a non-significant correlation between the AUC of treosulfan and age (correlation coefficient: 0.178, *p*-value: 0.509). The data is summarized in Figure 1.

### 3.6. Progression-Free Survival and Overall Survival

The PFS and OS rates at 12 months for the BuMel group were 56% and 75%, respectively. For the TreoMel group, the PFS at 12 months was 50% and the OS was 80%. The median follow-up was longer in the BuMel group as compared to the TreoMel group (36.5 versus 23 months; *p* = 0.0089). The rate of relapsing patients did not differ between both groups (BuMel: 44%; TreoMel: 55%; *p* = 0.738), with a median interval from ASCT to relapse of 6 months in both cohorts (*p* = 0.547).

**Figure 2:**
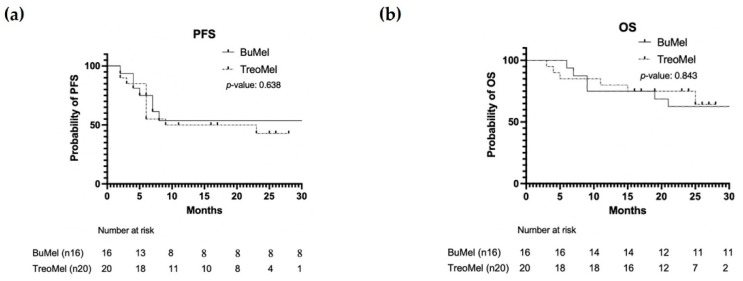
(**a**) Progression-free survival and (**b**) overall survival of both treatment groups.

The rate of death was 38% for BuMel patients and 30% for TreoMel patients (*p* = 0.730), with a median time from ASCT to death of 9 and 8 months, respectively (*p* = 0.766). These data are shown in Table 2 and Figure 2.

## 4. Discussion

So far, there is no standardized pre-ASCT conditioning regimen in AML patients. In the past, a combination of either BuMel or BuCy was used [10]. Recently, treosulfan, a prodrug of a bifunctional alkylating agent, previously used in ovarian cancer, has been investigated as a conditioning regimen before allogeneic stem cell transplantation [19,21,24,30,33]. Nevertheless, so far, no study has investigated the use of treosulfan for HDCT/ASCT. Treosulfan was described to offer a better safety profile compared to busulfan, without compromising the effects of treatment [33]. Another concern regarding busulfan is the high interpatient variability of the bioavailability of the drug, leading to compulsory therapeutic drug monitoring, which may not be of necessity when patients are treated with treosulfan [16,30,31,32].

In this study, we analyzed the combination of treosulfan plus melphalan compared to the combination of busulfan with melphalan regarding toxicity of treatment and interpatient variability in plasma concentration of the drugs, as well as treatment outcomes in patients with AML undergoing ASCT.

First, in our study, the combination of treosulfan and melphalan offered a good safety profile, with few serious adverse events. Of special mention was the complete absence of central nervous system toxicities in the TreoMel cohort, whereas encephalopathy and seizures were documented in the BuMel cohort in two (13%) and one (6%) patients, respectively. These differences may be explained by the inability of treosulfan and its epoxides to penetrate the mature blood–brain barrier as documented in the rat model [34,35].

Irreversible total or partial alopecia was experienced by 44% of patients in the BuMel cohort. In contrast, permanent alopecia did not occur in the TreoMel cohort (*p* = 0.0014).

A common toxicity of busulfan reported in many previous studies is veno-occlusive disease [11,12,16]. In our study, we found no cases of veno-occlusive disease (VOD) in either cohort. Nevertheless, a reversible liver enzyme increase was observed in almost all patients in both cohorts.

Plasma concentrations of treosulfan measured in this study using liquid chromatography–mass spectrometry were lower than the mean levels described in the literature. A comparison of various plasma concentrations as suggested by different researchers is listed in Table 5. This incoherence may be due to the variable age of the participants in the different studies. Most past studies focused on a pediatric patient population. Children do not tend to have fully developed kidneys, leading to treosulfan accumulation [34]. Additionally, we could identify a considerable interpatient variability in the plasma concentrations of treosulfan in our cohort, which emphasizes the need for therapeutic drug monitoring, including for treosulfan. Several reasons for the high interpatient variability of treosulfan were suggested in the literature. The impact of parameters such as body surface area (BSA), body weight, height, age, and renal function, as well as the use of diuretics have been evaluated as possible reasons for the interpatient variability of the volume of distribution at steady state and total clearance (and, consequently, AUC). Only BSA has been found to influence the volume of the central compartment, with a higher BSA leading to a higher clearance of the drug [36].

We found a low correlation between higher patient age (and, thus, body composition) and higher AUC of treosulfan, which may be due to the limited cohort size. Additionally, we observed a low correlation between the AUC of treosulfan and the amount of grade II–IV AEs, but significance was not reached. In a pediatric cohort with various diseases including hemoglobinopathies, primary immune deficiencies, and different malignancies, amongst others, such associations have been described for treosulfan. Van der Stoep et al. suggested an AUC of over 1650 mg/L*h in children to be associated with a higher frequency of multiple organ toxicities compared to an AUC of below 1350 mg/L*h [25]. The authors thus recommended plasma concentration measurement for treosulfan for all pediatric patients.

Clinical outcomes in terms of PFS and OS were similar in both cohorts in our study, although the limited median follow-up in the TreoMel cohort and the limited cohort size had to be taken into account. We see this study as a starting point for investigating the combination of treosulfan and melphalan before ASCT in AML patients, and we recognize the need for further research into this promising therapy.

## 5. Conclusions

In conclusion, we found an absence of serious adverse events and similar clinical outcomes for patients in the TreoMel and BuMel cohorts. Treosulfan combined with melphalan seems to be a safe alternative to busulfan/melphalan for AML patients in the ASCT setting. Importantly, TreoMel, compared to BuMel, does not lead to central nervous toxicities or irreversible alopecia. While it had been postulated that therapeutic drug monitoring might not be necessary for treosulfan, we observed considerable interpatient variability in the plasma concentration of the drug. Therefore, therapeutic drug monitoring seems to be a sensible idea for patients under treosulfan HDCT. The results of the study are limited by the small patient number and shorter follow-up time. Thus, further investigations should be conducted to confirm the safety and outcome results of this treatment combination. Independent multicenter studies should confirm our results, aiming to further establish treosulfan in the HDCT/ASCT setting for AML patients.

## Figures and Tables

**Table 1:** Patient characteristics at diagnosis (BuMel *n* = 16, TreoMel *n* = 20).

Characteristics	BuMel (*n* = 16)	TreoMel (*n* = 20)	All Patients (*n* = 36)	*p*-Value
Mean age at diagnosis, years (range)	57 (38–73)	51 (33–72)	54 (34)	0.120
Males/females (ratio)	8/8 (1.0)	14/6 (2.3)	22/14 (1.6)	0.667
ELN risk categories, favorable	8	9	17	0.999
intermediate	5	10	15	0.492
adverse	3	1	4	0.292
FAB classification, M0	2 (13%)	1 (5%)	3 (8%)	0.574
M1	6 (38%)	4 (20%)	10 (28%)	0.285
M2	2 (13%)	9 (45%)	11 (31%)	0.067
M4	3 (19%)	4 (20%)	7 (19%)	0.999
M5	2 (13%)	2 (10%)	4 (11%)	0.999
Secondary AML	1 (6%)	1 (5%)	2 (6%)	0.999
Peripheral Blood Parameters				
WBC, G/L	18 (±24)	41 (±78)	30 (±61)	0.264
Platelets, G/L	89 (±79)	77 (±57)	82 (±67)	0.603
Hemoglobin, g/dL	92 (±28)	89 (±23)	90 (±24)	0.735
Peripheral blasts, %	37 (±30)	43 (±26)	43 (±27)	0.285
BM blasts, %	72 (±24)	76 (±20)	75 (±22)	0.621
LDH, U/L	956 (±834)	889 (±531)	938 (±705)	0.065

BuMel: busulfan + melphalan patient cohort, TreoMel: treosulfan + melphalan patient cohort, ELN: European LeukemiaNet risk stratification, FAB: French-American-British classification, AML: acute myeloid leukemia, WBC: white blood cell, BM: bone marrow, LDH: lactate dehydrogenase.

**Table 2:** Details of engraftment and clinical outcomes.

Parameter	BuMel (*n* = 16)	TreoMel (*n* = 20)	All Patients (*n* = 36)	*p*-Value
Median follow up, months (range)	36.5 (6–48)	23 (3–28)	23.5 (3–48)	0.0089
Median time from diagnosis to ASCT, months (range)	4 (2–13)	3 (2–6)	3 (2–13)	0.164
Median CD34+ cells at ASCT, *n* × 10^6^ /kg b.w. (range)	4.12 (2.46–8.12)	3.86 (2.85–5.94)	4.03 (2.46–8.12)	0.788
Median time to neutrophil recovery, days (range)	12 (11–33)	12 (11–21)	12 (11–33)	0.417 (ns)
Median time to neutropenia, days (range)	5 (2–7)	5 (2–8)	5 (2–8)	0.567 (ns)
Median time in neutropenia, days (range)	7 (5–28)	8 (5–11)	7.5 (5–28)	0.507 (ns)
Median time to platelet recovery, days (range)	23 (13–137)	23 (11–89)	36 (11–137)	0.221 (ns)
Median hospitalization duration, days (range)	25 (21–39)	20 (18–101)	24 (18–101)	0.574 (ns)
Relapse, *n* (%)	7 (44%)	11 (55%)	18 (50%)	0.738 (ns)
Median interval to relapse, months (range)	6 (2–8)	6 (2–23)	6 (1–23)	0.547 (ns)
Deaths, number (%)	6 (38%)	6 (30%)	12 (33%)	0.730 (ns)
Median time to death, months (range)	9 (6–21)	8 (3–25)	9 (3–25)	0.766 (ns)

BuMel: busulfan + melphalan patient cohort, TreoMel: treosulfan + melphalan patient cohort, ns: not significant.

**Table 3:** Overview of infections in both treatment groups.

Parameter	BuMel (*n* = 16)	TreoMel (*n* = 20)	*p*-Value
Febrile episode	16 (100%)	20 (100%)	0.999
Causative agent identified	12 (75%)	14 (70%)	0.999
Bacterial agent identified	12 (75%)	13 (65%)	0.718
Viral agent identified	3 (19%)	2 (10%)	0.632
Fungal agent identified	1 (7%)	1 (5%)	0.999
Multiple infectious foci identified	4 (25%)	1 (5%)	0.141

BuMel: busulfan + melphalan patient cohort, TreoMel: treosulfan + melphalan patient cohort.

**Table 4:** Important toxicities.

Parameter	Grade I		Grade II		Grade III		Grade IV		*p*-Value
Toxicity, *n* (%)	Bu Mel *n* = 16	Treo Mel *n* = 20	Bu Mel *n* = 16	Treo Mel *n* = 20	Bu Mel *n* = 16	Treo Mel *n* = 20	Bu Mel *n* = 16	Treo Mel *n* = 20	
Diarrhea	8 (50)	6 (30)	0 (0)	4 (20)	4 (25)	8 (40)	0 (0)	0 (0)	0.374
Mucositis	5 (31)	7 (35)	1 (6)	2 (10)	3 (19)	3 (15)	0 (0)	0 (0)	0.999
Irreversible alopecia	3 (19)	0 (0)	4 (25)	0 (0)	0 (0)	0 (0)	0 (0)	0 (0)	0.0014
Partial	3 (19)	0 (0)	-	-	-	-	-	-	0.078
Complete	-	-	4 (25)	0 (0)	-	-	-	-	0.031
Increase of liver enzymes	7 (44)	37 (5)	4 (25)	7 (35)	4 (25)	4 (20)	1 (6)	0 (0)	0.492
Veno-occlusive disease	0 (0)	0 (0)	0 (0)	0 (0)	0 (0)	0 (0)	0 (0)	0 (0)	0.999
Epileptic seizure	0 (0)	0 (0)	1 (6)	0 (0)	0 (0)	0 (0)	0 (0)	0 (0)	0.444
Encephalopathy	1 (6)	0 (0)	1 (6)	0 (0)	0 (0)	0 (0)	0 (0)	0 (0)	0.191
Thrush	2 (13)	8 (40)	1 (6)	0 (0)	2 (13)	0 (0)	0 (0)	0 (0)	0.731
Engraftment syndrome	0 (0)	0 (0)	0 (0)	0 (0)	5 (31)	4 (20)	0 (0)	0 (0)	0.470

BuMel: busulfan + melphalan patient cohort, TreoMel: treosulfan + melphalan patient cohort.

**Table 5:** Summary of treosulfan plasma measurements found in the literature, all with doses of 3 × 14 g/m^2^.

Reference	Number of Patients	Median Age (Years)	AUC, μg/mL*h (Mean ± SD)
Mohanan et al. [30]	87	9	1396 ± 715
Sender et al. [31]	10	51	1104 ± 173
Nemecek et al. [16]	12	34	1309 ± 262
Baronciani et al. [32]	7	7.5	2400 ± 1267
Present study	20	50.9	836.79 ± 117.27

## Data Availability

The data presented in this study are available on request from the corresponding author.

## Supplementary Materials

The following supporting information can be downloaded at: https://www.mdpi.com/article/10.3390/cancers14041024/s1, Figure S1: Flowchart visualizing the inclusion of patients; Table S1: Cytogenetic and molecular abnormalities at diagnosis; Table S2: Overview of all toxicities experienced by patients in both cohorts.
