# Supplementary material for: Comparison of Melphalan Combined with Treosulfan or Busulfan as High-Dose Chemotherapy before Autologous Stem Cell Transplantation in AML
Source: Cancers (Basel). 2022 Feb 17;14(4):1024. doi: 10.3390/cancers14041024 (PMC8869805; doi:10.3390/cancers14041024)
Supplement: Supplementary file 1 [file cancers-14-01024-s001.zip › cancers-1555506-supplementary.pdf]

Supplementary Materials

Comparison of Melphalan Combined with Treosulfan or Busulfan as High-Dose Chemotherapy before Autologous Stem Cell Transplantation in AML

Ekaterina Gurevich, Michael Hayoz, Yolanda Aebi, Carlo R. Largiadèr, Behrouz Mansouri Taleghani, Ulrike Bacher and Thomas Pabst

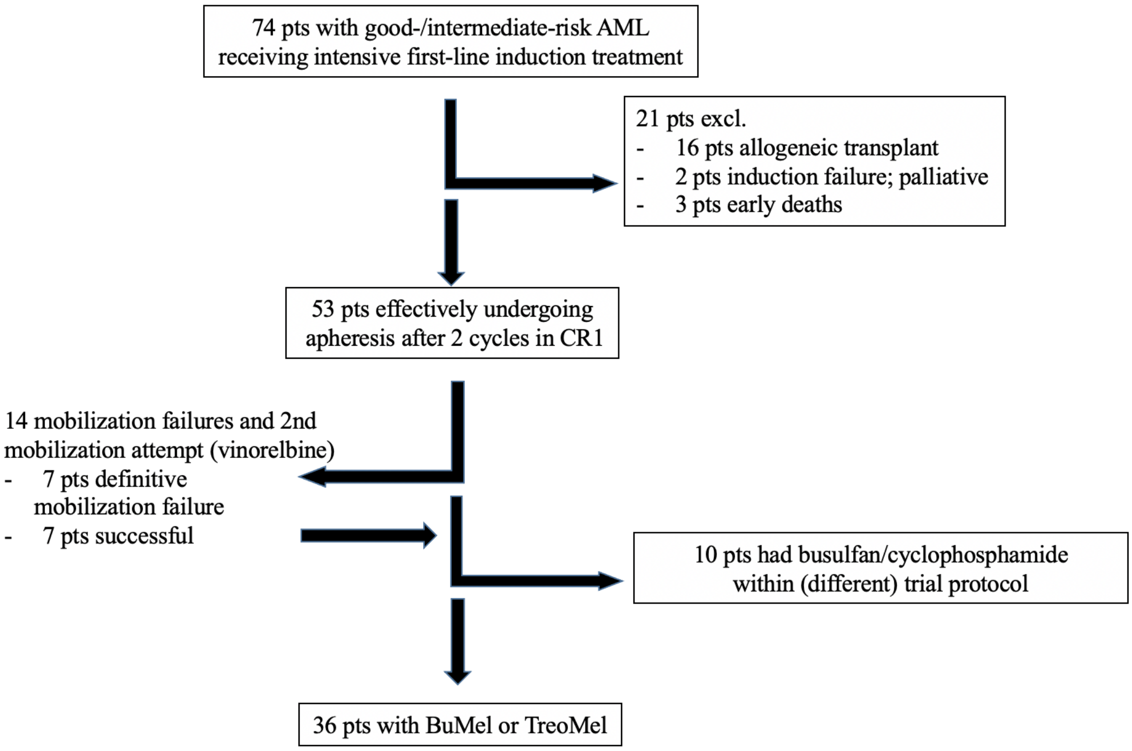

Figure S1. Flowchart visualizing the inclusion of patients. BuMel: Busulfan + Melphalan patient cohort, TreoMel: Treosulfan + Melphalan patient cohort, AML: Acute Myeloid Leukemia, CR: Complete Remission, Pts: Patients, excl.: excluded.

Table S1. Cytogenetic and molecular abnormalities at diagnosis (BuMel n = 16, TreoMel n = 20).

Genetic Abnormalities	BuMel (n = 16)	TreoMel (n = 20)	All patients (n = 36)	p-value
Karyotypes				
Normal karyotype	13 (81%)	12 (60%)	25 (70)	0.277
t(9;11)(p21:q23)	0 (0%)	1 (5%)	1 (3%)	0.999
t(8;21)(q22;q22)	0 (0%)	2 (10%)	2 (6%)	0.492
inv(16)(p13.1q22)	1 (6%)	3 (15%)	4 (11%)	0.613
del(9q)	1 (6%)	0 (0%)	1 (3%)	0.999
Independent clones	0 (0%)	1 (5%)	1 (3%)	0.999
Complex karyotype	1 (5%)	1 (5%)	2 (6%)	0.999
Molecular aberrations				
AML1-ETO (*RUNX1/RUNXT1*)	0 (0%)	2 (10%)	2 (6%)	0.492
ASXL1	1 (6%)	0 (0%)	1 (3%)	0.999
BCOR	1 (6%)	0 (0%)	1 (3%)	0.999

BCR-ABL1	1 (6%)	0 (0%)	1 (3%)	0.999
CBL	1 (6%)	0 (0%)	1 (3%)	0.999
CEBPA	1 (6%)	1 (5%)	2 (6%)	0.999
c-KIT	1 (6%)	2 (10%)	3 (8%)	0.999
DNMT3A	3 (19%)	2 (10%)	5 (14%)	0.637
ETV6	0 (0%)	1 (5%)	1 (3%)	0.999
FLT3-ITD	3 (19%)	4 (20%)	7 (20%)	0.999
FLT3-TKD	2 (13%)	4 (20%)	6 (17%)	0.672
IDH1	1 (6%)	0 (0%)	1 (3%)	0.999
IDH2	5 (31%)	6 (30%)	11 (31%)	0.999
inv(16)/*CBFB-MYH11*	1 (6%)	3 (15%)	4 (11%)	0.613
KIT D816V	1 (6%)	0 (0%)	1 (3%)	0.999
KRAS	0 (0%)	1 (5%)	1 (3%)	0.999
MLL (KMT2A) rearrangement	0 (0%)	1 (5%)	1 (3%)	0.999
NF1	0 (0%)	2 (10%)	2 (6%)	0.492
NPM1 mutation	8 (50%)	8 (40%)	16 (44%)	0.737
NRAS	0 (0%)	2 (10%)	2 (6%)	0.492
PHF6	0 (0%)	1 (5%)	1 (3%)	0.999
RUNX1	2 (13%)	1 (5%)	3 (8%)	0.574
SRSF1	1 (6%)	0 (0%)	1 (3%)	0.999
SRSF2	1 (6%)	2 (10%)	3 (8%)	0.999
TET2	3 (19%)	1 (5%)	4 (11%)	0.303
TP53	0 (0%)	1 (5%)	1 (3%)	0.999

BuMel: Busulfan + Melphalan patient cohort, TreoMel: Treosulfan + Melphalan patient cohort.

Table S2. Overview of all toxicities experienced by patients in both cohorts.

System Organ Class (SOC)		Grade I		Grade II		Grade III		Grade IV		*p*
		BuMel *n* = 16 (%)	TreoMel *n* = 20 (%)	BuMel *n* = 16 (%)	TreoMel *n* = 20 (%)	BuMel *n* = 16 (%)	TreoMel *n* = 20 (%)	BuMel *n* = 16 (%)	TreoMel *n* = 20 (%)	
Metabolism and nutrition disorders	Decreased appetite	18.75	10	6.25	40	75	50	0	0	0.999
	Electrolyte imbalance	56.25	85	31.25	15	12.5	0	0	0	0.999
	Hyperglycemia	12.5	5	81.25	90	0	0	0	0	0.999
Gastrointestinal disorders	Diarrhea	50	30	0	20	25	40	0	0	0.374
	Vomiting	81.25	55	0	5	0	0	0	0	0.277
	Nausea	87.5	50	0	20	0	0	0	0	0.257
	Mucositis	31.25	35	6.25	10	18.75	15	0	0	0.999
	Constipation	68.75	55	0	10	0	0	0	0	0.999
	Colitis (neutropenic)	0	15	31.25	40	0	0	0	0	0.191
	Abdominal pain	31.25	30	12.5	10	0	0	0	0	0.999
	Dysphagia	6.25	5	6.25	0	0	5	0	0	0.999
	Gastrointestinal Reflux	37.5	0	0	0	0	0	0	0	0.004
General disorders and administration site conditions	Fatigue	81.25	55	12.5	0	0	0	0	0	0.022
	Edema limbs	50	45	0	0	0	0	0	0	0.999
	Non-cardiac chest pain	6.25	0	0	10	0	5	0	0	0.613
Renal/urinary disorders	Acute Kidney Injury	0	0	0	0	12.5	5	0	0	0.574
Nervous system disorders	Headache	12.5	15	0	5	0	0	0	0	0.672
	Peripheral sensory neuropathy	12.5	5	0	0	0	0	0	0	0.574
	Seizure	0	0	6.25	0	0	0	0	0	0.444
	Dysgeusia	43.75	35	0	0	0	0	0	0	0.734
	Dizziness	25	20	6.25	0	0	0	0	0	0.470
	Tremor	18.75	10	0	0	0	0	0	0	0.637
	Encephalopathy	6.25	0	6.25	0	0	0	0	0	0.191
Respiratory, thoracic, and mediastinal disorders	Oropharyngeal Pain	62.5	40	25	15	0	10	0	0	0.245
	Epistaxis	0	0	0	0	43.75	15	0	0	0.073
	Hiccups	0	5	6.25	5	6.25	0	0	0	0.999
Infections	Thrush	12.5	40	6.25	0	12.5	0	0	0	0.731
	Pharyngitis	0	0	25	20	0	0	0	0	0.999
	Bacteremia	0	0	43.75	35	0	0	0	0	0.734
	Sepsis	0	0	0	0	0	0	0	5	0.999
	Lung infection	0	0	6.25	5	6.25	0	0	5	0.999
	Catheter-site infections	0	0	12.5	5	12.5	0	0	0	0.149
	Skin infection	18.75	0	0	0	0	0	0	0	0.078
	Vaginal infection	6.25	0	0	0	0	0	0	0	0.444
Skin and subcutaneous disorders	Purpura	87.5	70	0	0	0	0	0	0	0.246

	Irreversible alopecia	18.75	0	25	0	0	0	0	0	0.0014
	Rash maculo-papular	6.25	20	0	10	0	0	0	0	0.104
	Pruritus	12.5	30	0	0	0	0	0	0	0.257
	Nail loss	6.25	5	25	0	0	0	0	0	0.069
	Hyperhidrosis	18.75	10	0	0	0	0	0	0	0.637
Laboratory parameters	Liver enzymes increased	43.75	35	25	35	25	20	6.25	0	0.492
	Blood LDH increased	62.5	40	0	0	0	0	0	0	0.315
	Hyperuricemia	6.25	10	0	0	0	0	0	0	0.999
Musculoskeletal and connective tissue disorders	Bone Pain	43.75	45	0	0	0	0	0	0	0.999
Vascular disorders	Hypotension	43.75	20	0	6.67	0	0	0	0	0.082
	Hypertension	6.25	10	0	15	0	0	0	0	0.196
	Thromboembolic event	0	0	6.25	0	0	0	0	0	0.444
Psychiatric disorders	Insomnia	18.75	20	0	0	0	0	0	0	0.999
Eye disorders	Dry eye	0	0	6.25	0	0	0	0	0	0.444
	Floaters	6.25	0	0	0	0	0	0	0	0.444
Hepatobiliary disorders	Ocular icterus	6.25	10	0	0	0	0	0	0	0.999
	Veno-occlusive disease	0	0	0	0	0	0	0	0	0.999
Cardiac disorders	Sinus Tachycardia	6.25	0	6.25	0	0	0	0	0	0.191
	Sinus Bradycardia	6.25	0	0	0	0	0	0	0	0.444
Immune system disorders	Others: Engraftment Syndrome	0	0	0	0	31.25	20	0	0	0.470
Injury, poisoning, and procedural complications	Graft failure	0	0	0	0	5	0	0	0	0.999

BuMel: Busulfan + Melphalan patient cohort, TreoMel: Treosulfan + Melphalan patient cohort.
